# *N*′-(4-Cyano­benzyl­idene)thio­phene-2-carbohydrazide

**DOI:** 10.1107/S1600536810019215

**Published:** 2010-05-26

**Authors:** Yu-Feng Li, Fu-Gong Zhang, Fang-Fang Jian

**Affiliations:** a Microscale Science Institute, Department of Chemistry and Chemical Engineering, Weifang University, Weifang 261061, People’s Republic of China; b Ministry of Personnel, Weifang University, Weifang 261061, People’s Republic of China; c Microscale Science Institute, Weifang University, Weifang 261061, People’s Republic of China

## Abstract

The title compound, C_13_H_9_N_3_OS, was prepared by the reaction of thio­phene-2-carbohydrazide and 4-formyl­benzonitrile. The dihedral angle between the benzene and thio­phene rings is 11.9 (1)°. In the crystal structure, mol­ecules are linked into centrosymmetric dimers by pairs of N—H⋯O hydrogen bonds.

## Related literature

For related structures, see: Girgis (2006 ▶); Jiang (2010 ▶).
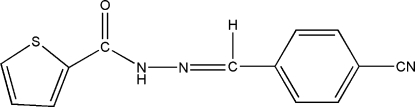


## Experimental

### 

#### Crystal data

C_13_H_9_N_3_OS
*M* *_r_* = 255.29
Monoclinic, 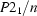

*a* = 6.3966 (13) Å
*b* = 16.340 (3) Å
*c* = 11.494 (2) Å
β = 90.66 (3)°
*V* = 1201.3 (4) Å^3^
*Z* = 4
Mo *K*α radiation
μ = 0.26 mm^−1^
*T* = 293 K
0.21 × 0.20 × 0.18 mm

#### Data collection

Bruker SMART CCD diffractometer
11589 measured reflections
2746 independent reflections
1539 reflections with *I* > 2σ(*I*)
*R*_int_ = 0.066

#### Refinement

*R*[*F* ^2^ > 2σ(*F* ^2^)] = 0.046
*wR*(*F* ^2^) = 0.145
*S* = 1.03
2746 reflections
163 parameters
H-atom parameters constrained
Δρ_max_ = 0.23 e Å^−3^
Δρ_min_ = −0.32 e Å^−3^

### 

Data collection: *SMART* (Bruker, 1997 ▶); cell refinement: *SAINT* (Bruker, 1997 ▶); data reduction: *SAINT*; program(s) used to solve structure: *SHELXS97* (Sheldrick, 2008 ▶); program(s) used to refine structure: *SHELXL97* (Sheldrick, 2008 ▶); molecular graphics: *SHELXTL* (Sheldrick, 2008 ▶); software used to prepare material for publication: *SHELXTL*.

## Supplementary Material

Crystal structure: contains datablocks global, I. DOI: 10.1107/S1600536810019215/lh5046sup1.cif

Structure factors: contains datablocks I. DOI: 10.1107/S1600536810019215/lh5046Isup2.hkl

Additional supplementary materials:  crystallographic information; 3D view; checkCIF report

## Figures and Tables

**Table 1:** Hydrogen-bond geometry (Å, °)

*D*—H⋯*A*	*D*—H	H⋯*A*	*D*⋯*A*	*D*—H⋯*A*
N2—H2*A*⋯O1^i^	0.86	1.97	2.821 (2)	171

Symmetry code: (i) 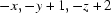.

**Fig. 1.**
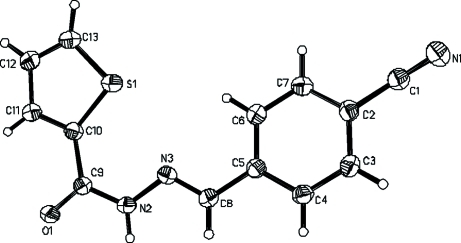
The structure of the title compound showing 30% probability displacement ellipsoids and the atom-numbering scheme.

## supplementary crystallographic information

### Comment

General background references for the title compound are included in the publication of a related structure (Jiang, 2010). As part of our search for new schiff base compounds we synthesized the title compound (I), and describe its structure here.

The molcular structure of (I) is shown in Fig. 1. The C8═N3 bond length of 1.272 (3)Å is comparable with reported values [1.281 (2) Å] (Girgis, 2006) and [1.273 (3)Å] (Jiang, 2010). In the crystal structure, molecules are linked by intermolecular N—H···O hydrogen bonds into centrosymmetric dimers.

### Experimental

A mixture of the thiophene-2-carbohydrazide (0.10 mol), and 4-formylbenzonitrile (0.10 mol) was stirred in refluxing ethanol (10 mL) for 4 h to afford the title compound (0.079 mol, yield 79%). Single crystals suitable for X-ray measurements were obtained by recrystallization from ethanol at room temperature.

### Refinement

H atoms were fixed geometrically and allowed to ride on their attached atoms, with C—H distances = 0.93-0.97 Å; N—H = 0.86Å and with *U*_iso_(H) = 1.2*U*_eq_(C,*N*) or 1.5*U*_eq_(C_methyl_).

### Crystal data

**Table:**

C_13_H_9_N_3_OS	*F*(000) = 528
*M**_r_* = 255.29	*D*_x_ = 1.412 Mg m^−^^3^
Monoclinic, *P*2_1_/*n*	Mo *K*α radiation, λ = 0.71073 Å
Hall symbol: -P 2yn	Cell parameters from 1539 reflections
*a* = 6.3966 (13) Å	θ = 3.8–26.2°
*b* = 16.340 (3) Å	µ = 0.26 mm^−^^1^
*c* = 11.494 (2) Å	*T* = 293 K
β = 90.66 (3)°	Block, colorless
*V* = 1201.3 (4) Å^3^	0.21 × 0.20 × 0.18 mm
*Z* = 4	

### Data collection

**Table:**

Bruker SMART CCD diffractometer	1539 reflections with *I* > 2σ(*I*)
Radiation source: fine-focus sealed tube	*R*_int_ = 0.066
graphite	θ_max_ = 27.5°, θ_min_ = 3.1°
φ and ω scans	*h* = −8→8
11589 measured reflections	*k* = −21→21
2746 independent reflections	*l* = −14→14

### Refinement

**Table:**

Refinement on *F*^2^	Primary atom site location: structure-invariant direct methods
Least-squares matrix: full	Secondary atom site location: difference Fourier map
*R*[*F*^2^ > 2σ(*F*^2^)] = 0.046	Hydrogen site location: inferred from neighbouring sites
*wR*(*F*^2^) = 0.145	H-atom parameters constrained
*S* = 1.03	*w* = 1/[σ^2^(*F*_o_^2^) + (0.0682*P*)^2^] where *P* = (*F*_o_^2^ + 2*F*_c_^2^)/3
2746 reflections	(Δ/σ)_max_ < 0.001
163 parameters	Δρ_max_ = 0.23 e Å^−^^3^
0 restraints	Δρ_min_ = −0.31 e Å^−^^3^

### Special details

**Table:**

Geometry. All esds (except the esd in the dihedral angle between two l.s. planes) are estimated using the full covariance matrix. The cell esds are taken into account individually in the estimation of esds in distances, angles and torsion angles; correlations between esds in cell parameters are only used when they are defined by crystal symmetry. An approximate (isotropic) treatment of cell esds is used for estimating esds involving l.s. planes.
Refinement. Refinement of *F*^2^ against ALL reflections. The weighted *R*-factor wR and goodness of fit *S* are based on *F*^2^, conventional *R*-factors *R* are based on *F*, with *F* set to zero for negative *F*^2^. The threshold expression of *F*^2^ > σ(*F*^2^) is used only for calculating *R*-factors(gt) etc. and is not relevant to the choice of reflections for refinement. *R*-factors based on *F*^2^ are statistically about twice as large as those based on *F*, and *R*- factors based on ALL data will be even larger.

### Fractional atomic coordinates and isotropic or equivalent isotropic displacement parameters (Å^2^)

**Table:**

	*x*	*y*	*z*	*U*_iso_*/*U*_eq_	
N3	0.2706 (3)	0.38738 (12)	0.84452 (15)	0.0428 (5)	
N2	0.1294 (3)	0.44128 (12)	0.88894 (14)	0.0440 (5)	
H2A	0.1499	0.4603	0.9579	0.053*	
O1	−0.1694 (3)	0.51089 (11)	0.87605 (12)	0.0542 (5)	
C2	0.8862 (4)	0.20156 (14)	0.79598 (18)	0.0444 (6)	
C9	−0.0408 (4)	0.46559 (14)	0.82840 (17)	0.0421 (6)	
C4	0.7596 (4)	0.29587 (15)	0.93785 (18)	0.0463 (6)	
H4A	0.7759	0.3224	1.0089	0.056*	
C8	0.4290 (4)	0.37040 (14)	0.90796 (18)	0.0430 (6)	
H8A	0.4457	0.3960	0.9797	0.052*	
C7	0.7082 (4)	0.21428 (16)	0.72907 (19)	0.0535 (7)	
H7A	0.6895	0.1858	0.6597	0.064*	
C5	0.5847 (4)	0.31123 (13)	0.86961 (17)	0.0408 (6)	
C10	−0.0772 (4)	0.43914 (14)	0.70730 (17)	0.0431 (6)	
C3	0.9102 (4)	0.24155 (15)	0.90156 (19)	0.0487 (6)	
H3B	1.0278	0.2318	0.9479	0.058*	
C13	−0.0822 (5)	0.38914 (16)	0.5070 (2)	0.0600 (8)	
H13A	−0.0568	0.3664	0.4344	0.072*	
C6	0.5585 (4)	0.26912 (15)	0.76500 (19)	0.0505 (6)	
H6A	0.4397	0.2780	0.7193	0.061*	
C11	−0.2581 (4)	0.45925 (15)	0.64795 (18)	0.0514 (7)	
H11A	−0.3673	0.4888	0.6802	0.062*	
C12	−0.2588 (5)	0.43005 (16)	0.5330 (2)	0.0595 (7)	
H12A	−0.3688	0.4380	0.4806	0.071*	
S1	0.08838 (11)	0.38350 (4)	0.62050 (5)	0.0558 (3)	
N1	1.1789 (4)	0.10803 (16)	0.7206 (2)	0.0703 (7)	
C1	1.0483 (4)	0.14800 (17)	0.7545 (2)	0.0516 (6)	

### Atomic displacement parameters (Å^2^)

**Table:**

	*U*^11^	*U*^22^	*U*^33^	*U*^12^	*U*^13^	*U*^23^
N3	0.0461 (13)	0.0436 (12)	0.0388 (9)	0.0034 (10)	0.0026 (9)	−0.0032 (8)
N2	0.0477 (13)	0.0492 (12)	0.0351 (9)	0.0055 (10)	−0.0021 (9)	−0.0080 (8)
O1	0.0571 (12)	0.0630 (12)	0.0425 (8)	0.0186 (9)	−0.0023 (8)	−0.0126 (8)
C2	0.0460 (15)	0.0440 (14)	0.0432 (11)	0.0022 (11)	0.0034 (11)	0.0055 (10)
C9	0.0471 (15)	0.0414 (13)	0.0376 (11)	0.0018 (11)	−0.0021 (11)	−0.0001 (10)
C4	0.0514 (16)	0.0490 (14)	0.0384 (11)	−0.0003 (12)	−0.0013 (11)	−0.0017 (10)
C8	0.0459 (15)	0.0463 (14)	0.0369 (11)	−0.0016 (11)	−0.0012 (10)	0.0013 (10)
C7	0.0612 (18)	0.0539 (16)	0.0452 (12)	0.0049 (13)	−0.0047 (12)	−0.0094 (11)
C5	0.0435 (15)	0.0404 (13)	0.0385 (11)	−0.0002 (11)	0.0022 (10)	0.0036 (9)
C10	0.0507 (15)	0.0401 (13)	0.0386 (11)	0.0018 (11)	0.0006 (10)	−0.0024 (9)
C3	0.0464 (16)	0.0517 (15)	0.0478 (12)	0.0026 (12)	−0.0026 (11)	0.0065 (11)
C13	0.081 (2)	0.0600 (18)	0.0391 (12)	0.0101 (16)	−0.0040 (13)	−0.0079 (11)
C6	0.0496 (16)	0.0538 (16)	0.0478 (12)	0.0066 (13)	−0.0063 (11)	−0.0027 (11)
C11	0.0591 (18)	0.0528 (16)	0.0420 (12)	0.0071 (13)	−0.0077 (11)	−0.0061 (11)
C12	0.071 (2)	0.0596 (17)	0.0477 (13)	0.0082 (15)	−0.0166 (13)	−0.0056 (12)
S1	0.0620 (5)	0.0646 (5)	0.0407 (3)	0.0109 (3)	−0.0001 (3)	−0.0088 (3)
N1	0.0686 (18)	0.0765 (17)	0.0658 (14)	0.0177 (14)	0.0068 (13)	−0.0044 (12)
C1	0.0534 (17)	0.0544 (16)	0.0471 (13)	0.0029 (13)	0.0009 (12)	0.0027 (12)

### Geometric parameters (Å, °)

**Table:**

N3—C8	1.272 (3)	C7—C6	1.378 (3)
N3—N2	1.365 (2)	C7—H7A	0.9300
N2—C9	1.345 (3)	C5—C6	1.394 (3)
N2—H2A	0.8600	C10—C11	1.376 (3)
O1—C9	1.239 (3)	C10—S1	1.723 (2)
C2—C7	1.382 (3)	C3—H3B	0.9300
C2—C3	1.385 (3)	C13—C12	1.349 (4)
C2—C1	1.442 (4)	C13—S1	1.693 (3)
C9—C10	1.474 (3)	C13—H13A	0.9300
C4—C3	1.378 (3)	C6—H6A	0.9300
C4—C5	1.382 (3)	C11—C12	1.405 (3)
C4—H4A	0.9300	C11—H11A	0.9300
C8—C5	1.460 (3)	C12—H12A	0.9300
C8—H8A	0.9300	N1—C1	1.133 (3)
			
C8—N3—N2	116.90 (18)	C6—C5—C8	120.8 (2)
C9—N2—N3	122.15 (18)	C11—C10—C9	121.4 (2)
C9—N2—H2A	118.9	C11—C10—S1	110.98 (16)
N3—N2—H2A	118.9	C9—C10—S1	127.61 (19)
C7—C2—C3	119.9 (2)	C4—C3—C2	119.9 (2)
C7—C2—C1	119.9 (2)	C4—C3—H3B	120.1
C3—C2—C1	120.2 (2)	C2—C3—H3B	120.1
O1—C9—N2	119.03 (19)	C12—C13—S1	112.96 (18)
O1—C9—C10	119.6 (2)	C12—C13—H13A	123.5
N2—C9—C10	121.3 (2)	S1—C13—H13A	123.5
C3—C4—C5	120.6 (2)	C7—C6—C5	120.1 (2)
C3—C4—H4A	119.7	C7—C6—H6A	119.9
C5—C4—H4A	119.7	C5—C6—H6A	119.9
N3—C8—C5	120.9 (2)	C10—C11—C12	112.2 (2)
N3—C8—H8A	119.6	C10—C11—H11A	123.9
C5—C8—H8A	119.6	C12—C11—H11A	123.9
C6—C7—C2	120.1 (2)	C13—C12—C11	112.5 (3)
C6—C7—H7A	119.9	C13—C12—H12A	123.7
C2—C7—H7A	119.9	C11—C12—H12A	123.7
C4—C5—C6	119.2 (2)	C13—S1—C10	91.29 (13)
C4—C5—C8	120.0 (2)	N1—C1—C2	177.8 (3)

### Hydrogen-bond geometry (Å, °)

**Table:**

*D*—H···*A*	*D*—H	H···*A*	*D*···*A*	*D*—H···*A*
N2—H2A···O1^i^	0.86	1.97	2.821 (2)	171

Symmetry codes: (i) −*x*, −*y*+1, −*z*+2.
